# Comparison of Prices for Commonly Administered Drugs in Employer-Sponsored Insurance Relative to Medicare

**DOI:** 10.1001/jamahealthforum.2022.5422

**Published:** 2023-02-10

**Authors:** Jessica Y. Chang, Aditi P. Sen

**Affiliations:** 1 Health Care Cost Institute, Washington, DC

## Abstract

This cross-sectional study compares unit prices and price growth in employer-sponsored insurance and Medicare over 2016 through 2020 for physician-administered drugs with the highest use and those with the highest spending.

## Introduction

Prices for physician-administered drugs among people with employer-sponsored insurance (ESI) have risen substantially over time, threatening access to lifesaving medication.^1^ Previous work has shown that ESI prices for cancer drugs are higher than prices for the same drugs in Medicare but has not examined prices of other physician-administered drugs or relative growth in prices over time.^2^ Unlike Medicare, where physician-administered drug prices are based on average sales price (ASP), ESI prices are negotiated between health care professionals or health systems and insurers. In this study, we compare unit prices and price growth in ESI and Medicare over 2016 through 2020 for physician-administered drugs with the highest use and those with the highest spending.

## Methods

We obtained data for 2016 through 2020 from Medicare ASP files and Health Care Cost Institute (HCCI) statistically deidentified ESI claims data.^3,4^ We identified the 10 drugs with the highest spending and use in the HCCI data in 2016 through 2020. We calculated ESI and Medicare unit prices for each drug, which allowed for direct comparison across payers and mitigated concerns about varying dosages (eg, by clinical indication). We limited the HCCI data to professional and noninpatient facility claims and identified drugs using the Healthcare Common Procedure Coding System (HCPCS). We defined ESI unit price as the median total allowed amount divided by total units for each patient-service date for each drug and year.^2^ We defined Medicare unit price as the average of quarterly ASP for a given HCPCS-year plus 6%. The primary outcome was the unit price percentage difference (markup) between ESI and Medicare rates by HCPCS-year. We examined characteristics of top drugs to identify features associated with high markups. See eMethods in Supplement 1 for additional detail. This study followed the STROBE reporting guidelines for cross-sectional studies.

## Results

Among the top spend drugs in 2020, the highest ESI price markups over Medicare were for pegfilgrastim (54%), trastuzumab (33%), nivolumab (24%), pertuzumab (22%), and rituximab (20%) (Table 1). In contrast, prices of bevacizumab, natalizumab, and vedolizumab were similar in ESI and Medicare. Price markups increased between 2016 and 2020 for 5 of the top 10 spend drugs and more than doubled for 3 (pegfilgrastim, trastuzumab, and rituximab).

**Table 1.** ESI and Medicare Unit Prices and ESI Price Markup of Top Administered Drugs^a^

Top drug	2016			2020		
	Unit price, $		Markup, %	Unit price, $		Markup, %
	ESI	Medicare^b^		ESI	Medicare^b^	
By spend						
Infliximab (Remicade)	97.5	86.6	13	65.8	56.1	17
Pegfilgrastim (Neulasta)	5212.5	4174.4	25	6355.9	4137.6	54
Trastuzumab (Herceptin)	111.4	96.2	16	144.8	109.2	33
Bevacizumab (Avastin)	75.8	75.9	0	82.9	83.5	−1
Rituximab (Rituxan)^c^	904.1	827.7	9	1122.9	936.3	20
Vedolizumab (Entyvio)	19.0	18.2	4	21.8	21.6	1
Nivolumab (Opdivo)	33.0	27.2	21	37.1	30.1	23
Natalizumab (Tysabri)	19.7	18.5	6	22.5	22.5	0
Pertuzumab (Perjeta)	13.7	11.2	22	16.6	13.6	22
Immune globulin	45.6	42.7	7	45.1	43.7	3
By use						
Ondansetron	2.7	0.1	2351	2.2	0.1	2155
Ketorolac tromethamine	2.4	0.7	237	2.5	0.6	307
Triamcinolone acetonide	1.9	1.9	2	1.6	1.6	1
Dexamethasone	0.4	0.1	200	0.5	0.1	280
Fentanyl citrate	7.3	0.5	1334	6.3	0.9	583
Ceftriaxone sodium	1.0	0.6	49	0.8	0.6	40
Normal saline solution	19.0	2.1	824	15.6	2.7	473
Propofol	0.9	0.1	606	0.7	0.1	519
Midazolam	4.1	0.1	2833	4.1	0.1	3350
Methylprednisolone acetate	4.9	5.0	−2	6.3	6.4	−2

Abbreviation: ESI, employer-sponsored insurance.

^a^ Top spend and top use are identified and ranked based on spending and use (number of claims) in the 2019 Health Care Cost Institute ESI claims data set.

^b^ Medicare unit price is based on average sales price. Prices are for 1 unit of drug; drug dosage is generally more than 1 unit and may vary by clinical indication and patient weight.

^c^ A 2018 change in the Healthcare Common Procedure Coding System resulted in different units of reimbursement for rituximab. Prices were standardized to pre-2018 unit reimbursement.

Unit prices were substantially lower for nearly all of the top 10 drugs by use compared with the top spend drugs; however, ESI markups were higher. The ESI plans paid more than 30 times as much as Medicare per unit of midazolam and more than 20 times as much for ondansetron in 2020. Five other top use drugs had ESI prices more than 200% higher than Medicare prices (fentanyl citrate, propofol, saline solution, ketorolac tromethamine, dexamethasone). The ESI markups were not clearly associated with the presence of biosimilars or approval year (Table 2).

**Table 2.** Market and Other Characteristics of Top Administered Drugs^a^

Top drug	FDA approval year	Unit per price, mg	No. of approved biosimilars (No. launched)	Approved drug therapeutic areas	Total HCCI	
					Spending, $ millions	Claims
By spend						
Infliximab (Remicade)	1998	10	4 (3)	Antipsoriatics, DMARDs, TNF inhibitors, immunosuppressants, monoclonal antibodies, inflammatory bowel disease agents	7008	1 072 713
Pegfilgrastim (Neulasta)	2002	6	4 (3)	Hematopoietic growth factors	3749	498 715
Trastuzumab (Herceptin)	1998	10	5 (5)	Antineoplastics, anti-*ERBB2* therapies, monoclonal antibodies	3336	455 737
Bevacizumab (Avastin)	2004	10	2 (2)	Antineoplastics, VEGF inhibitors, monoclonal antibodies	2653	611 240
Rituximab (Rituxan)	1997	10	3 (2)	DMARDs, antineoplastics, anti-CD20 monoclonal antibodies	1960	181 199
Vedolizumab (Entyvio)	2014	1	0	Inflammatory bowel disease agents, monoclonal antibodies, integrin blockers	1938	246 335
Nivolumab (Opdivo)	2014	1	0	Antineoplastics, PD-1/PD-L1 inhibitors	1763	128 649
Natalizumab (Tysabri)	2004	1	0	Multiple sclerosis treatments, inflammatory bowel disease agents, monoclonal antibodies, integrin blockers	1745	224 896
Pertuzumab (Perjeta)	2012	1	0	Antineoplastics	1605	174 724
Immune globulin	2005	500	NA	Immune globulins	1198	216 451
By use						
Ondansetron	1997	1	NA	Antiemetics (selective 5-HT3 antagonists)	276	8 664 335
Ketorolac tromethamine	1997	15	NA	NSAIDs	165	8 392 500
Triamcinolone acetonide	2007	10	NA	Corticosteroids	86	8 241 782
Dexamethasone	1958	1	NA	Corticosteroids, anti-inflammatory agents	148	8 148 964
Fentanyl citrate	1968	0.1	NA	Opioid analgesics	175	6 287 922
Ceftriaxone sodium	1982	250	NA	Cephalosporins	140	5 458 916
Saline solution	1970	1000 cc	NA	NA	257	5 205 693
Propofol	1989	10	NA	General anesthetics	235	5 070 624
Midazolam	1985	1	NA	Antianxiety agents, anxiolytics, benzodiazepines	124	5 068 958
Methylprednisolone acetate	1957	40	NA	Corticosteroids, anti-inflammatory agents	45	4 427 658

Abbreviations: DMARD, disease-modifying antirheumatic drug; FDA, US Food and Drug Administration; HCCI, Health Care Cost Institute; NA, not applicable; NSAID, nonsteroidal anti-inflammatory drug; PD-1, programmed cell death 1; PD-L1, PD-1 ligand 1; TNF, tumor necrosis factor; VEGF, vascular endothelial growth factor.

^a^ Information on unit of reimbursement was sourced from Medicare average sales price files, information on number of biosimilars (approved and launched) was sourced from the Center for Biosimilars at the *American Journal of Managed Care*, information on drugs’ clinical indications was sourced from searches on Medscape, and total unweighted employer-sponsored insurance spending and use were sourced from the authors’ analyses of HCCI data from 2016 through 2020.

## Discussion

In this cross-sectional study, we found that ESI plans paid more than Medicare for most top spend and top use physician-administered drugs, including those with biosimilars available. While high-volume drugs had relatively low unit prices, ESI markups over Medicare prices were especially high for these drugs. A limitation of this analysis is that ESI unit price was derived from claims and may not equal actual negotiated price. Furthermore, other research has found different prices across sites of care (office vs outpatient); we do not account for this variation.^5^ The present data set is drawn from 3 insurers, and findings may not be generalizable to other health plans.

High ESI prices for physician-administered drugs raise overall spending and threaten access to innovative and lifesaving products for close to half of the US population with health insurance through their employer. Policies to reduce ESI prices for physician-administered drugs will be important; these efforts are needed for both low-price, commonly used drugs and the most expensive drugs.

## Notes

Supplement 1. eMethods

Supplement 2. Data Sharing Statement
