# Supplementary material for: Comparison of Prices for Commonly Administered Drugs in Employer-Sponsored Insurance Relative to Medicare
Source: JAMA Health Forum. 2023 Feb 10;4(2):e225422. doi: 10.1001/jamahealthforum.2022.5422 (PMC9918879; doi:10.1001/jamahealthforum.2022.5422)
Supplement: Supplement 1. — eMethods [file jamahealthforum-e225422-s001.pdf]

Supplemental Online Content

Chang JY, Sen AP. Comparison of prices for commonly administered drugs in employer-sponsored insurance relative to Medicare. *JAMA Health Forum*. 2023;4(2):e225422. doi:10.1001/jamahealthforum.2022.5422

eMethods

This supplemental material has been provided by the authors to give readers additional information about their work.

© 2022 Chang JY et al. *JAMA Health Forum.*

eMethods

DATA

Our study used data from the Health Care Cost Institute (HCCI) that included claims of 55 million enrollees with employer-sponsored health plans from Aetna, Humana, and over 30 Blue Cross Blue Shield plans dating back to 2012. Collectively, the data cover one-third of people with employer-sponsored health plans in the U.S. across the 50 states, and the District of Columbia. HCCI claims data include allowed amounts, the actual prices paid by health plans and patients, as well as procedure codes, diagnosis codes, and enrollee information (age category, subscriber status, sex, 5-digit zip code of residence). The data also include encrypted patient and provider identifiers.

KEY VARIABLES: UNIT PRICE AND EMPLOYER-SPONSORED INSURANCE MARKUP

The primary variable in this research letter was the unit price for injectable and infusion drugs administered in outpatient and physician office settings in the HCCI data, i.e., among individuals with employer-sponsored health insurance coverage. Unit prices were used in order to directly compare to administered drug prices in employer-sponsored insurance to Medicare's listed ASP unit prices.

There is no field for providers to complete in the CMS-1500 (physician) and UB-04 (facility) claim forms that indicates negotiated unit price. Therefore, we calculated the estimate unit price for each administered drug based on the allowed amounts and bill unit for each service date. Price, defined as total allowed amounts divided by total use, is a definition that Health Care Cost Institute (HCCI) has used for multiple reports such as Health Care Cost Utilization Report. Furthermore, the use of unit price to measure drug price is found in other literature such as Xiao et al. (2022).

This research letter opted to use unit-based price to circumvent data issues with differential dosing which could arise if our outcome relied on dose-based price (i.e., total allowed amount for the administered drug per service day). Drug dosages often vary by clinical indication for drugs with multiple approved indications; for example, there is a large dosage difference in bevacizumab for treatment of macular degeneration compared to dosage for treatment of cancer. Additionally, dosage also differs for patients initiating therapy (e.g., starter doses) and subsequent titration to higher doses.

We opted to calculate the relative price difference (i.e., percent difference) between employer-sponsored insurance and Medicare unit prices by drug-year instead of using the absolute dollar differences. While absolute dollar difference between employer-sponsored insurance and Medicare can be more salient to interpret, we opted to standardize the price levels between high priced drugs in the high spend category and low priced drugs in high use category by presenting relative price markups. Absolute dollar differences between unit prices do not fully

© 2022 Chang JY et al. *JAMA Health Forum.*

capture the difference in physician revenues between Medicare and employer-sponsored insurance administration of these drugs since payment rate is also dependent on dosing which may differ across the two populations.

DRUG SAMPLE

In this research letter, we limited our study to the top 10 drugs (identified by HCPCS code) by spending (defined as total allowed amount) and use (defined as total claims) in the HCCI data between 2016 and 2020. We included both top spend and top use drugs to provide a more complete look at markups across these different types of administered drugs. The top spend drugs are drugs that are lower use among the population with employer-sponsored insurance but with far higher prices. On the other hand, top use drugs consist of very common and lower priced drugs that are typically used during a variety of health care services (e.g., outpatient infusion and emergency room department).

Our unit of observation was at the HCPCS level. Importantly, each product (biologic originator and each biosimilar) has its own HCPCS code for billing purposes. For example, Remicade (trade name for originator infliximab) has its own HCPCS code of J1745. Each infliximab biosimilar drug product has a corresponding HCPCS (e.g., Q5103 for Inflectra, Q5104 for Renflexis, Q5109 for Ixifi, and Q5121 for Avsola). This allows us to directly compare unit prices at a product level between employer-sponsored insurance and Medicare and abstract away from concerns regarding the use of brand and generic/biosimilar drugs across payers.

© 2022 Chang JY et al. *JAMA Health Forum.*
