# Supplementary material for: Comparison of Prices for Commonly Administered Drugs in Employer-Sponsored Insurance Relative to Medicare
Source: JAMA Health Forum. 2023 Feb 10;4(2):e225422. doi: 10.1001/jamahealthforum.2022.5422 (PMC9918879; doi:10.1001/jamahealthforum.2022.5422)
Supplement: Supplement 2. — Data Sharing Statement [file jamahealthforum-e225422-s002.pdf]

Data Sharing Statement

Chang. Comparison of Prices for Commonly Administered Drugs in Employer-Sponsored Insurance Relative to Medicare. *JAMA Health Forum*. Published February 10, 2023. doi:10.1001/jamahealthforum.2022.5422

Data
Data available: No

Additional Information
Explanation for why data not available: HCCI's data use agreement does not allow us to share individual-level claims.
